# Mix Design and Performance Study of High-Strength Self-Compacting Concrete with Manufactured Sand

**DOI:** 10.3390/ma18010055

**Published:** 2024-12-26

**Authors:** Xuan Liu, Xuhao Wang, Yuan Wang, Qianqian Liu, Yuan Tian, Jie Zhou, Yahong Meng

**Affiliations:** 1 School of Highway, Chang’an University, Xi’an 710064, China; liu.xuan@chd.edu.cn (X.L.); wangyuan2022@chd.edu.cn (Y.W.); qqliu@chd.edu.cn (Q.L.); tianyuan198346@163.com (Y.T.); 2 School of Construction Engineering Management, Inner Mongolia Technical College of Construction, Hohhot 010070, China; 3 Xizang Gaozheng Building Materials Company, Lhasa 851400, China; zhoujie1113@yeah.net; 4 Gansu Yuanlong Road and Bridge Mechanized Highway Engineering Co., Ltd., Lanzhou 730070, China

**Keywords:** self-compacting concrete, manufactured sand, concrete design mix, workability, compressive strength

## Abstract

In recent years, research on self-compacting concrete (SCC) has gradually shifted towards high-strength development, while high-strength self-compacting concrete has been widely used in applications such as precast bridge components and high-rise building projects. Using manufactured sand as an aggregate can effectively address the challenges posed by the depletion of natural sand resources. This study optimized the mix design for high-strength self-compacting concrete with manufactured sand (MSH-SCC) and explored the effects of the fine aggregate replacement rate, sand ratio, and maximum particle size of coarse aggregate on the performance of MSH-SCC. The results indicated that the optimized mix designs for various strength levels met the performance requirements. The fine aggregate replacement rate and the maximum nominal aggregate size significantly affected the workability of the concrete, while variations in the sand ratio had a smaller impact. The yield stress of the MSH-SCC showed a positive correlation with the fine aggregate replacement rate and the maximum nominal aggregate size, whereas the plastic viscosity reached its maximum value under specific conditions. Additionally, the mix design parameters had a limited effect on the mechanical strength of the MSH-SCC. This study provides a scientific basis for the design of high-strength self-compacting concrete with manufactured sand, contributing to the promotion of manufactured sand use and advancing low-carbon development in the construction industry.

## 1. Introduction

Self-compacting concrete (SCC) can completely fill molds under its own weight, effectively reducing the difficulty of vibrating concrete in building structures with a large amount of reinforcement [1,2,3,4]. It has been widely used in engineering [5].

The design methods for SCC mix proportions can be mainly categorized into the following types: (a) Design methods for SCC mix proportions based on the fixed aggregate content [6]. This design approach was proposed by Okamura [7,8], which involves limiting the aggregate content, using a lower water-to-cement ratio, and employing high-performance water-reducing agents in trial mixes until the desired workability is achieved [9]. (b) Design methods for SCC mix proportions based on the aggregate packing density factors [9,10,11,12]. The basic principle of this method is to treat the cementitious materials and the aggregate system separately, viewing the mix design as a process of filling the voids between loosely packed aggregates with the cementitious paste. The aggregate packing density factor (PF) is selected based on the target workability of the SCC, and the amounts of coarse and fine aggregates are determined [10,11]. Based on the design strength of the SCC, the amounts of cement, water-to-cement ratio, mineral admixtures, and high-performance water-reducing agents are calculated sequentially [12]. (c) Design methods for SCC mix proportions based on empirical parameters. Based on empirical data, the unit aggregate quantity, cementitious material quantity, and water-to-cement ratio are determined through experience formulae summarized in engineering practice [13,14]. Currently, the Chinese technical specification for the application of SCC, as well as Japanese standards and European guidelines, also follow the basic idea of the empirical trial mix method for designing SCC mix proportions. (d) Design methods for SCC mix proportions based on statistical factors. Represented by Khayat [13] and Sonebi [14], statistical factors are used for the experimental design of SCC mix proportions and the prediction of the related performance [15,16]. Raw material component parameters are treated as independent variable factors, while the test results are treated as dependent variables [17]. A functional relationship between the SCC mix proportion component parameters and test results is established to calculate the mix proportions.

In general, there are differences in the design methods and standards for SCC mix proportions, and a lack of unified standards and specifications among different mix design methods. More importantly, mix design methods are mainly aimed at low-strength SCC [18], and there is a relative lack of research on the mix design for high-strength self-compacting concrete (HS-SCC) [19]. The engineering application of HS-SCC lacks references. In the past few decades, particularly in countries with a high construction demand like China and India, the design methods and cases for SCC mix proportions have primarily been focused on medium- and low-strength SCC with strength grades below C60 [20]. There are relatively few cases of using manufactured sand to prepare SCC with strength grades reaching to C60 or even C80 [21]. With the continuous development of infrastructure construction, the demand for high-strength self-compacting concrete in structures such as precast bridge components, high-rise buildings, and hydraulic facilities has been steadily increasing [22]. Existing mix design methods are no longer able to meet the requirements of practical production.

Additionally, SCC is typically made with river sand. However, as high-quality, non-renewable river sand (RS) resources are gradually depleting [23], there is an increasing call for manufactured sand (MS) to replace natural sand as a fine aggregate in concrete, presenting a good opportunity for the concrete industry to move towards green and low-carbon development [24,25]. However, SCC has relatively strict requirements for fine aggregates [26]. Although the production of manufactured sand has largely achieved standardization and scalability, the manufactured sand produced from parent rock through processes such as crushing and dust removal still cannot meet the quality standards of high-quality medium sand in terms of the grading, particle shape, angularity, stone powder content, and MB value [25,27]. On the one hand, the stone powder of MS can improve the cohesiveness and water retention of the mixture, improve the interface performance of the concrete [28], optimize the particle size distribution of the concrete, and improve the compactness. The rough surface of manufactured sand increases the friction between the aggregates [29], facilitating the formation of an interlocking structure, thus enhancing the mechanical properties of the MS-SCC [30]. These characteristics of manufactured sand, however, can significantly reduce the rheology and workability of the SCC, regardless of the rise in the paste matrix, which is beneficial for lowering the flow resistance related to aggregate contacts and frictions [31]. High-strength concrete, with its low water–binder ratio and large dosage of water-reducing admixtures, struggles to maintain good workability. The rough surface of manufactured sand often significantly exacerbates the deterioration of its filling ability. Zhang [32] showed that MS greatly decreased the stability of SCC, and the static stability index increased from 10.3% to 29.13% when the amount of MS exceeded 20%, thus increasing risk of segregation. D. Suriya [33] prepared C50 MS-SCC and also reached the conclusion that the fluidity of the concrete deteriorates with the increase in manufactured sand. Therefore, how to decrease the adverse impacts of manufactured sand on the workability of SCC is one of the key issues in the application of high-strength self-compacting concrete with manufactured sand (MSH-SCC) [34].

Given the lack of a systematic mix design method and performance studies for MSH-SCC, this study replaced part of the river sand with manufactured sand and optimized the SCC mix designs for C40, C60, and C80 concretes, leading to a versatile method for producing high-strength concrete. At the same time, conventional concrete performance tests were conducted to verify the applicability of the optimized mix proportions. And key mix design parameters were also adjusted, for instance, the replacement rate of the manufactured sand (20, 40, and 60%), the sand ratio (47, 49, and 51% for C40 concrete; 45, 47, and 49% for C60 concrete; 39, 41, and 43% for C80 concrete), and the maximum nominal aggregate size (13, 20, and 25 mm), so to explore the variations in the workability and mechanical strength of MSH-SCC.

## 2. Materials and Methods

### 2.1. Raw Materials

Portland cement P·I 52.5 was provided by Ningguo Cement Plant (Xuancheng, China). S95 mineral powder was purchased from Ningbo Henglong Building Materials Technology Co., Ltd. (Ningbo, China). Class I fly ash was provided by Taizhou Power Plant (Taizhou, China). SF-92 silicon fume was purchased from Qinghai Lantian Environmental Protection Co., Ltd. (Xining, China). The chemical compositions of these materials are listed in Table 1.

**Figure 1:**
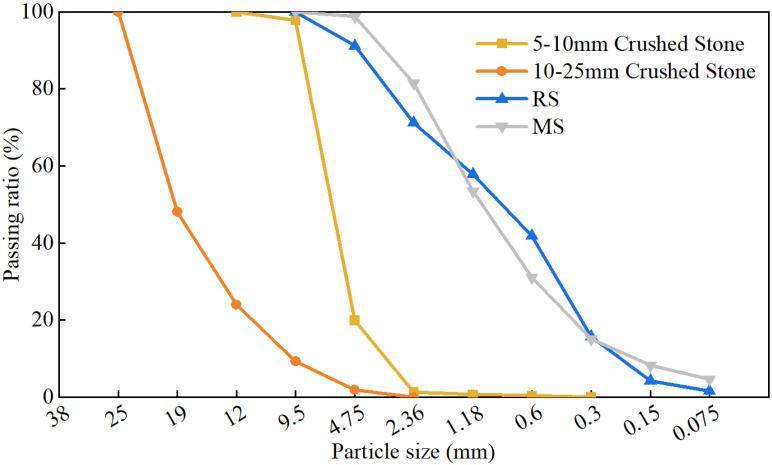
Particle size distribution of the aggregates.

The coarse aggregate used in the experiments was limestone, primarily divided into the following two sizes: 5–10 mm and 10–25 mm. The fine aggregates used consisted of the following two types: river sand and manufactured sand from tuff. Table 2 and Table 3, respectively, present the technical specifications for the fine and coarse aggregates, and Figure 1 illustrates the grading curve for the fine and coarse aggregates.

In addition, polycarboxylate superplasticizer (SP) was provided by Jiangsu Sobute New Material Co., Ltd. (Nanjing, China). The dosage of SP was determined based on the optimized mix proportions for the concrete at different strength levels.

### 2.2. Preparation of MSH-SCC

The cementitious material and dry sand aggregates were mixed for 150 s, and the SP was dissolved in water and then added into the mixture. After mixing for 120 s, the fresh MSH-SCC was obtained and used to test the workability and to fabricate the specimens.

The mixing was carried out under 20 ± 2 °C.

### 2.3. Workability Test

The workability of the SCC was carried out according to the Chinese standard for SCC [35]. The test used the slump flow, T500, and J-ring flow. Generally, a larger slump flow and shorter slump flow time indicate a better concrete filling performance. The difference between the values obtained from the J-ring flow test and the flow spread was referred to as the PA value; a smaller PA value indicates a better passing ability. The slump flow of the SCC should be the average of two diameters perpendicular to each other on the spread surface after the slump of the concrete mixture stops, measured with a precision of up to 1 mm, and with the results rounded to the nearest 5 mm. A stopwatch was used to measure the time that the concrete took for the slump flow to reach 500 mm, recorded as T500, with a precision of up to 0.1 s. The J-ring should be the average of two diameters perpendicular to each other on the spread surface after the slump of the concrete mixture stops, measured with a precision of up to 1 mm, and with the results rounded to the nearest 5 mm.

### 2.4. Rheological Test

**Figure 2:**
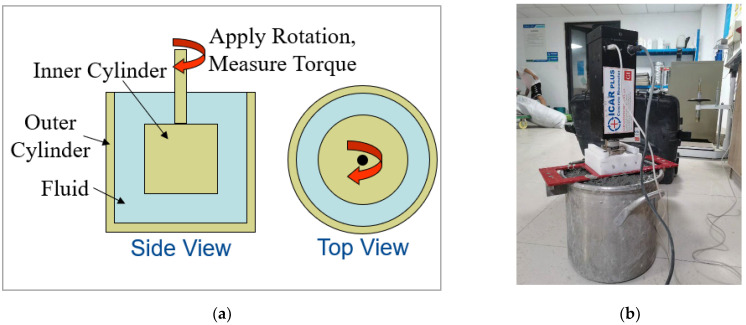
(**a**) Schematic diagram of a coaxial cylindrical rheometer. (**b**) Rheological test operation diagram.

The rheological properties of the concrete were measured using the ICAR coaxial cylinder rheometer, produced in Copenhagen, Denmark. This equipment consists of a computer, a testing bucket, a rheometer, and blades, as shown in Figure 2. Firstly, a stress growth test was conducted on the mixture at a low rotational speed. The mixture showed linear elastic behavior until reaching the yield torque, after which the internal structure was disrupted, causing a gradual decrease in torque. The peak torque value was then used to calculate the static yield stress. After the pre-shear, a flow curve test was performed, and the torque results from the descending segment were used to calculate the dynamic yield stress and plastic viscosity [36].

Many scholars, considering the characteristics of both Newtonian and non-Newtonian fluids, have proposed the Bingham fluid model, using the plastic viscosity and yield stress to characterize the rheological properties of concrete [37,38,39,40]. As shown in Equation (1), the model was represented by the Reiner–Riwlin equation:

(1) $$\begin{array}{l} \tau =\tau_{0}+\eta \frac{d\gamma }{dt} \end{array}$$

where τ (Pa) is the shear stress, τ_0_ (Pa) is the yield stress, H (Pa·s) is the plastic viscosity, and dγ/dt (s) is the shear rate.

(2) $$T_{S0}=\frac{2T_{m}}{\pi D^{3}(\frac{H}{D}+\frac{1}{3})}$$

where τ_s0_ (Pa) is the static yield stress, T_m_ (Pa) is the maximum yield torque, D (mm) is the diameter of the inner cylinder, and H (mm) is the height of the inner cylinder.

(3) T=G+HN

where T (Nm) is the torque in Newton-meters, N (rpa) is the rotational speed of the blade in revolutions per second, G is the intercept in the speed–torque relationship, and H is the slope in the speed–torque relationship.

(4) $$\tau_{0}=\frac{\left(\frac{1}{R_{1}^{2}}-\frac{1}{R_{2}^{2}}\right)}{\ln(\frac{R_{2}}{R_{1}})4\pi h}\times G$$

(5) $$\mu =\frac{\left(\frac{1}{R_{1}^{2}}-\frac{1}{R_{2}^{2}}\right)}{4\pi h}\times H$$

where τ (Pa) is the dynamic yield stress, R_1_ (mm) is the radius of the inner cylinder, R_2_ (mm) is the radius of the outer cylinder, G is the intercept, h (mm) is the height of the inner cylinder, μ is the plastic viscosity (Pa·s), and H is the slope.

### 2.5. Mechanical Properties Test

The fresh MSH-SCC was added into a formwork (100 mm × 100 mm× 100 mm) and demolded after 24 h in the curing room at 20 ± 1 °C and a relative humidity ≥ 95%; then, the samples were continually cured for 28 days. The 28-day compressive strength of the concrete specimens was carried out according to the concrete strength standards of China [41]. At least 3 sets of valid data were obtained for each test condition, and the average value was taken as the test result.

## 3. Optimization of Concrete Mix Design

### 3.1. Existing Concrete Mix Proportions

This study, based on the filling theory of cement paste and the theory of the paste excess coefficient, combined the aggregate system design method of the Tarantula Curve and the Power 45 chart to carry out the mix proportion design for the C40, C60, and C80 concretes.

Based on the preliminary experiments of the research group, the high-strength SCC had a low water-to-binder ratio and contained substantial cementitious materials, making the workability difficult to control. Moreover, an excess of manufactured sand increased the friction between the aggregates, significantly affecting the filling performance of the MSH-SCC, and the stone powder in the MS increased the viscosity of the paste, making self-compacting difficult to achieve [42]. To ensure the practical engineering requirements of the MSH-SCC, a replacement rate of 20% was chosen for the manufactured sand.

This study considered MSH-SCC as a multiphase system composed of an aggregate system and a paste system. Based on the original mix proportions of river sand self-compacting concrete from the research group, 20% of river sand was directly replaced with manufactured sand, and the mix proportions are shown in Table 4. In combination with the SCC design method by Brouwers and Radix, and utilizing the Marllon D. Cook’s Tarantula Curve and the Power 45 chart, the mix proportion of the aggregate system in the MSH-SCC was optimized through programming solutions to improve the continuity and compactness of the aggregate system [16,43,44]. This simplified the MSH-SCC mix design process.

### 3.2. Design of Aggregate System

**Figure 3:**
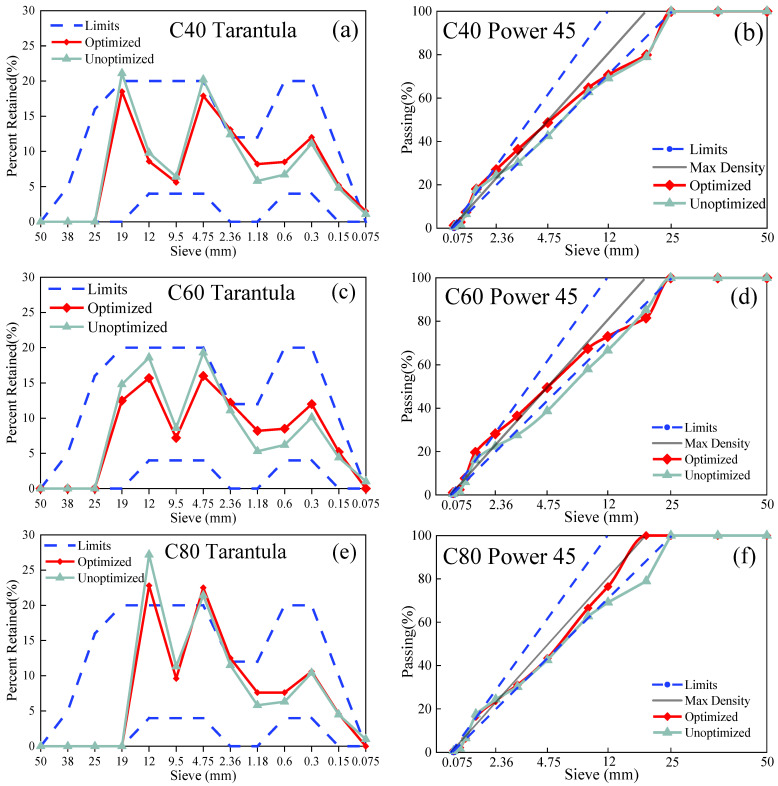
(**a**) Tarantula Curve for C40 concrete; (**b**) Power 45 chart for C40 concrete; (**c**) Tarantula Curve for C60 concrete; (**d**) Power 45 chart for C60 concrete; (**e**) Tarantula Curve for C80 concrete; (**f**) Power 45 chart for C80 concrete.

The Tarantula Curves of the aggregate system are illustrated in Figure 3a,c,e, showing the aggregate system before and after optimization. The Tarantula Curve, developed by Marllon D. Cook [16,45] at Oklahoma State University through an extensive analysis of experimental data, evaluated the mechanical and workability performance of the concrete prepared with different aggregate systems. Using the retained percentages of various sieve sizes as influencing factors, the curve defines the ideal retained percentage ranges for aggregates that yield high-performance concrete. The upper and lower limits of these ranged are plotted, and, because the curve resembles a tarantula, it was named the Tarantula Curve.

As depicted in Figure 3b,d,f, the aggregate system before and after optimization was illustrated by the Power 45 chart. The horizontal axis represents the Power 45 chart of the sieve aperture diameter, while the vertical axis indicates the percentage of the mass of each graded aggregate. The resulting curve distribution is known as the Power 45 chart. This curve can be used to study the particle size distribution characteristics of the aggregates. By observing the Power 45 chart, the continuity of the aggregate grading and proximity to the maximum density grading can be assessed. If the curve is relatively flat and close to the maximum density grading curve, it indicates that the aggregate grading is continuous and approaches the optimal state. Conversely, if the curve fluctuates significantly or deviates far from the maximum density grading curve, it suggests discontinuities or deviations in the aggregate grading.

Table 5 shows the aggregate components in the optimized composite system for three different strength grades, which aimed to prevent a sharp decline in the workability of the concrete due to a poor particle shape and grading of the manufactured sand. During the mix proportion optimization, a lower replacement rate of 20% manufactured sand was selected. Subsequently, the bulk density of the optimized graded aggregate system was measured, and the void ratio of the mixed aggregate system was calculated based on the computed bulk density values. Following this, the void volume of the concrete per cubic meter was determined based on the void ratio.

### 3.3. Design of Paste Composition

The design of the paste composition for the benchmark group in this study mainly references existing mature mix ratios. The composition and dosage of additives were improved to better meet the requirements of the SCC. The proportions of cementitious materials for different strength grades of concrete are shown in Table 6. The supplementary cementitious materials for C40 and C60 concretes are fly ash and mineral powder, while, for C80 concrete, the supplementary cementitious materials are silica fume and mineral powder. The reason for using silica fume instead of fly ash in the C80 concrete is that fly ash as a cementitious material is not conducive to the development of early strength in the concrete, whereas the inclusion of silica fume can significantly enhance the mechanical performance of the concrete [46,47].

### 3.4. Determination of Paste Volume

Using the theory of excess paste, the V_paste_/V_void_ was introduced to guide the design of the paste volume in the SCC. In this study, V_paste_ refers to the volume of the cement paste in one cubic meter of fresh concrete, while V_void_ represents the volume of voids in one cubic meter of fresh concrete. The V_paste_/V_void_ characterizes the amount of cement paste relative to the voids in the aggregate system. To ensure that the workability of each grade of concrete met the requirements, the selected V_paste_/V_void_ differed for various strength grades of cement concrete during the mix design process. In this study, the V_paste_/V_void_ was set based on the experience of Wang, X [48]. The V_paste_/V_void_ for the MSH-SCC at different strength grades is shown in Table 7.

### 3.5. Determination of Optimized Mix Design

The water–cement ratios for the C40, C60, and C80 strength grades were 0.40, 0.32, and 0.22, respectively. The sand ratios were 49%, 47%, and 41%. The replacement rate of the manufactured sand was 20%, and the maximum nominal particle size was 25 mm. Additionally, the appropriate amount of SP was selected based on the requirements of concrete of different grades. The mix proportions of the SCC containing 20% manufactured sand are presented in Table 8.

### 3.6. Analysis of Concrete Performance Before and After Mix Proportion Optimization

**Figure 4:**
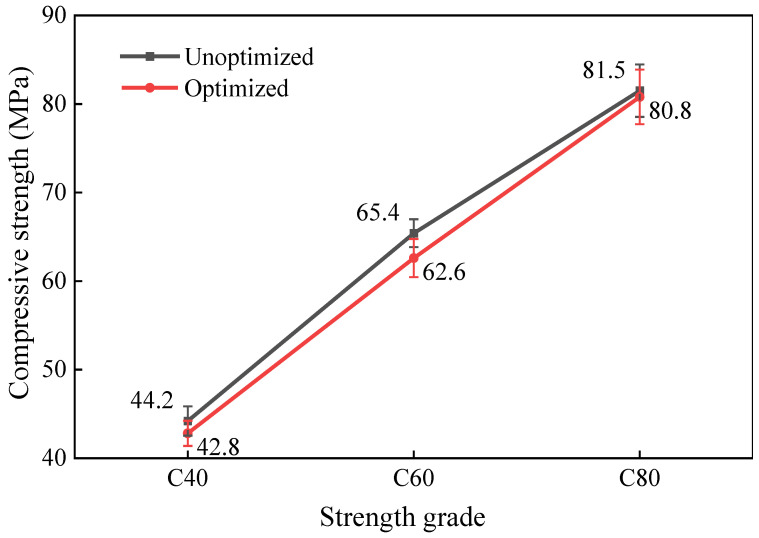
The 28-day compressive strength of the concrete with the original mix proportions and the optimized mix proportions.

To assess the effectiveness of the optimized mix proportion method, this study conducted a detailed comparative analysis of the performance of concrete before and after the mix proportion optimization under the same environmental conditions, and a fixed amount of water-reducing admixture. As shown in Table 9 and Figure 4, the workability and 28-day compressive strength of the C40, C60, and C80 concretes were compared before and after the optimization of the mix proportions. Under the unoptimized mix proportions, the 28-day compressive strength of the MSH-SCC was excellent, but it did not meet the requirements for self-compacting concrete. The optimized mix proportions resulted in a good workability for the MSH-SCC at all three strength levels, and the 28-day compressive strength met the design requirements.

Compared to the workability of the concrete with the original mix proportions, after optimization, the slump flow of the C40, C60, and C80 concretes increased by 6.1%, 3.4%, and 21.6%, respectively; the T500 were reduced by 1.4%, 11.5%, and 54.0%, respectively; the PA values were decreased by 6.7%, 5.3%, and 38.5%, respectively. To ensure the self-compacting performance of the MSH-SCC, this study designed an appropriate V_paste_/V_void_ based on the excess paste theory, ensuring that there was sufficient paste to envelop the aggregates in the MSH-SCC, and that the friction between the aggregates was reduced, thereby enhancing the workability.

Compared to the mechanical properties of the concrete with the initial mix proportions, after optimization, the 28-day compressive strength of the C40, C60, and C80 concretes slightly decreased by 3.2%, 4.3%, and 0.9%, respectively. Although there was a reduction in the strength, all met the targeted design strength. As the designed strength grade increases, the thickness of the paste between aggregates in the optimized concrete increases, which may lead to some loss of strength. However, this study optimized the aggregate system to reduce the porosity and improve the density of the concrete, ensuring the strength performance of the concrete to a certain extent.

## 4. Study on the Influence of Mix Proportion Parameters on the Performance of MSH-SCC

### 4.1. Workability

#### 4.1.1. Effect of Replacement Rate of Manufactured Sand on Concrete Workability

The replacement rate of the manufactured sand affected the workability of various strength grades, as shown in Table 10. The slump flow diameter showed a negative correlation with the substitution rate, while the T500 value generally exhibited a positive correlation. The PA value showed no significant change. Manufactured sand primarily impacted the fluidity of the concrete, with a minimal effect on the passing ability. The increase in the replacement rate of the manufactured sand influenced the overall angularity of the fine aggregates, the MB value, and the content of stone powder, which, in turn, increased the viscosity of the concrete mixture [49,50].

The higher the strength grade, the more sensitive the performance of the concrete is to the replacement rate of the manufactured sand. When the replacement rate of the manufactured sand increased from 20% to 60%, the slump spread of the C40, C60, and C80 concretes decreased by 8.2%, 9.8%, and 11.3%, respectively. Meanwhile, the T500 increased by 131.89%, 80%, and 100% in the same range. The T500 of the C80 concrete was significantly higher than that of the C60 concrete. In the C80 concrete, the addition of silica fume with a finer particle radius led to an increased demand for water in the cement paste. This modification, along with a reduced water–cement ratio and the incorporation of a higher dosage of water-reducing admixtures, resulted in the enhanced viscosity of the concrete. As a consequence, the T500 value for the C80 concrete was notably higher compared to that of the C60 concrete [51,52]. Therefore, the higher the target design strength, the more pronounced the effect of the manufactured sand replacement rate on the workability of the MSH-SCC.

**Figure 5:**
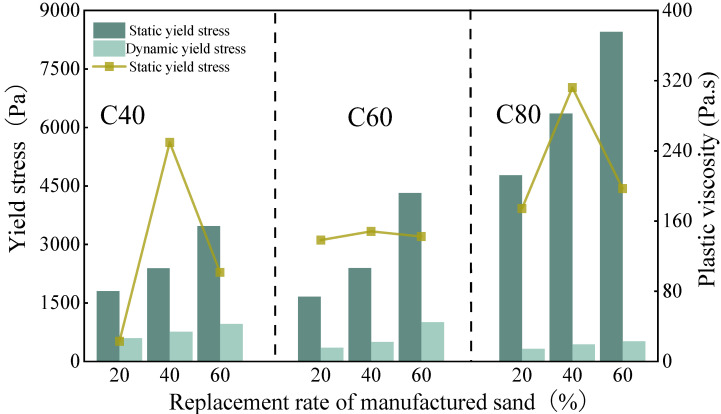
The effect of the manufactured sand replacement rate on the rheology of the concrete.

The influence of the proportion of the manufactured sand replacement rate on the rheology of the concrete is illustrated in Figure 5. The static and dynamic yield stresses of each concrete group showed a positive correlation with the replacement rate of the manufactured sand. The plastic viscosity initially increased with the rising replacement rate, but then it started to decrease. This behavior was also attributed to the differences in the particle shape and the surface roughness between the MS and NS [53]. The friction between the natural and manufactured sand led to a gradual increase in the plastic viscosity [54], but this effect reached a peak.

The low water–cement ratio and the addition of silica fume accelerated the early hydration process of the C80 concrete, which resulted in its static yield stress being significantly higher than that of the C40 and C60 concretes. This trend contrasted with the dynamic yield stress [55]. Although the C60 and C80 concretes had lower water–cement ratios, in the mix design of this study, the V_paste_/V_void_ ratio for the C60 and C80 concretes was higher, and the amount of SP was also greater than that of the C40 concrete. This partially offset the adverse effects of the water–cement ratio on the rheology of the C60 and C80 concretes.

#### 4.1.2. Effect of Sand Ratio on Concrete Workability

Table 11 demonstrates the impact of the sand ratio on the workability of the MSH-SCC across different strength levels. Within the specified range, the flowability of the concrete at various strength levels showed an upward trend with an increasing sand ratio. The variation in the sand ratio had a minimal impact on the gap permeability of the SCC. Increasing the sand ratio enhanced the volumetric proportion of the mortar, which played a crucial role in lubricating and filling the voids between the coarse aggregates in the concrete matrix. As the sand ratio rose, the frictional resistance between the coarse aggregates decreased, leading to improved workability. The impact of the sand ratio on the workability of the MSH-SCC was primarily focused on the flowability, with its effect on the gap permeability being negligible. Overall, the variations in the sand ratio had a limited influence on the workability of the MSH-SCC.

**Figure 6:**
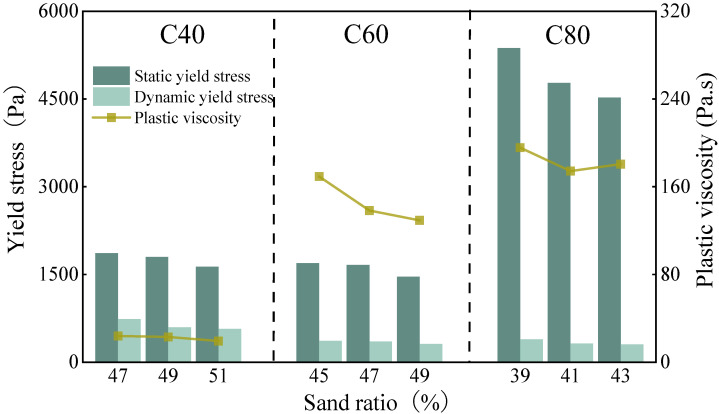
The effect of the sand ratio on the rheology of the concrete.

The influence of the sand ratio on the rheological properties of the MSH-SCC across different strength levels is illustrated in Figure 6. For the different strength levels of concrete, as the sand ratio increases, both the static yield stress and the dynamic yield stress increase, along with a rise in the plastic viscosity. This aligns with the influence of the sand ratio on the concrete workability.

#### 4.1.3. Effect of Maximum Nominal Aggregate Size on Concrete Workability

The influence of the maximum nominal aggregate size on the workability of the MSH-SCC at various strength grades is shown in Table 12. As the maximum nominal particle size of the aggregates in the concrete decreases, the flowability of the SCC improves, and the extent of its impact on the workability varies with the strength grade. When the maximum nominal particle size decreased from 25 mm to 13 mm, the slump spread of the three strength grades of the concrete increased by 3.4%, 6.2%, and 8.8%, respectively, while the T500 decreased by 0, 73%, and 50%. The C40 concrete was less affected compared to the C60 and C80 concretes due to its lower V_paste_/V_void_ ratio, indicating a weaker bond between the aggregate system and the paste system. As the maximum nominal particle size decreased, the changes in the passing ability and T500 became less pronounced. The PA values for all of the strength grades of the concrete increased with the maximum nominal particle size. Among the different strength grades, the C60 and C80 concretes exhibited a notable trend in the change in the PA values. When the maximum nominal particle size increased from 13 mm to 25 mm, the PA values increased by 25 mm and 20 mm, respectively. For all of the strength grades of the concrete, reducing the maximum nominal particle size significantly improved the workability, suggesting that the higher target strength designs were more susceptible to fluctuations in the maximum nominal particle size of the coarse aggregates.

**Figure 7:**
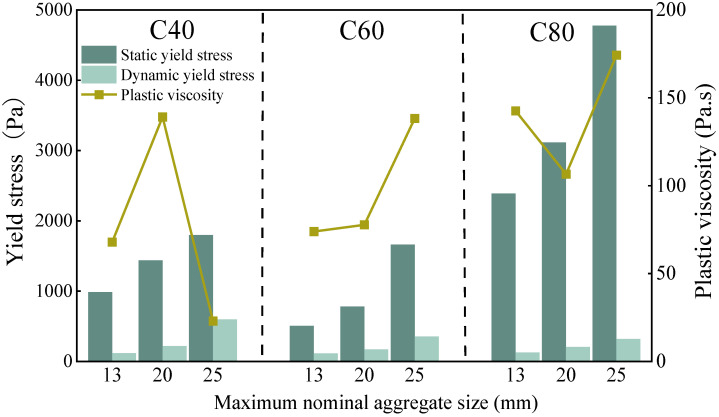
The effect of the maximum nominal aggregate size on the rheology of the concrete.

The influence of the maximum nominal particle size of the aggregates on the rheological properties of the MSH-SCC at various strength levels is illustrated in Figure 7. As the maximum nominal particle size increased, the yield stress also increased, although the rate of growth was relatively small, which is consistent with the previously mentioned workability indicators. With an increase in the average particle size of the aggregate system, the gaps between the concrete aggregate particles became larger [56]. The volume fraction of the smaller aggregates, which have good filling capacity, decreased, leading to an increase in the internal friction resistance within the system. The static yield stress of the C80 concrete was significantly higher than that of the C60 concrete, while the dynamic yield stress of the C60 concrete was notably greater than that of the C80 concrete. This can be attributed to the fact that an increase in the maximum nominal particle size reduces the internal voids and adhesion within the concrete, making it more difficult for the coarse aggregate particles to move and arrange themselves, thereby lowering the dynamic yield stress. No significant correlation exists between the plastic viscosity and the maximum nominal particle size; the MSH-SCC with a maximum particle size of 20 mm exhibits a lower plastic viscosity and demonstrates better rheological performance.

### 4.2. Compressive Strength

#### 4.2.1. Effect of Replacement Rate of Manufactured Sand on Concrete Compressive Strength

**Figure 8:**
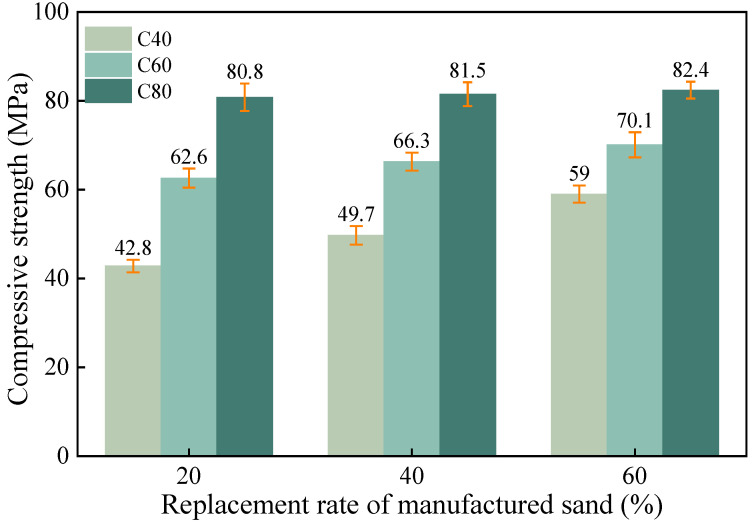
The effect of the replacement rate of the manufactured sand on the compressive strength of the concrete.

In Figure 8, the mechanical properties of the MSH-SCC show a positive correlation with the manufactured sand replacement rate. As the replacement rate increased from 20% to 60%, the 28-day strengths of the C40, C60, and C80 concretes increased by 37.9%, 12.0%, and 5.9%, respectively. This enhancement was attributed to the incorporation of manufactured sand, which improved the overall structural strength and the angularity of the fine aggregates in the concrete [57]. The static friction between the coarse and fine aggregates also increased, while the stone powder in the manufactured sand provided attachment points for cement hydration, promoting the formation of cement hydration products [58,59]. The higher the replacement rate of manufactured sand, the more pronounced this effect becomes. As the strength grade increased, the trend of the concrete strength improvement weakened with the increase in the replacement rate of the manufactured sand. The main reason for this phenomenon may be that the V_paste_/V_void_ ratio of the C40 concrete was lower than that of the C60 and C80 concretes, making its mechanical properties more sensitive to the increased roughness of the fine aggregates caused by the higher replacement rate of the manufactured sand.

#### 4.2.2. Effect of Sand Ratio on Concrete Compressive Strength

**Figure 9:**
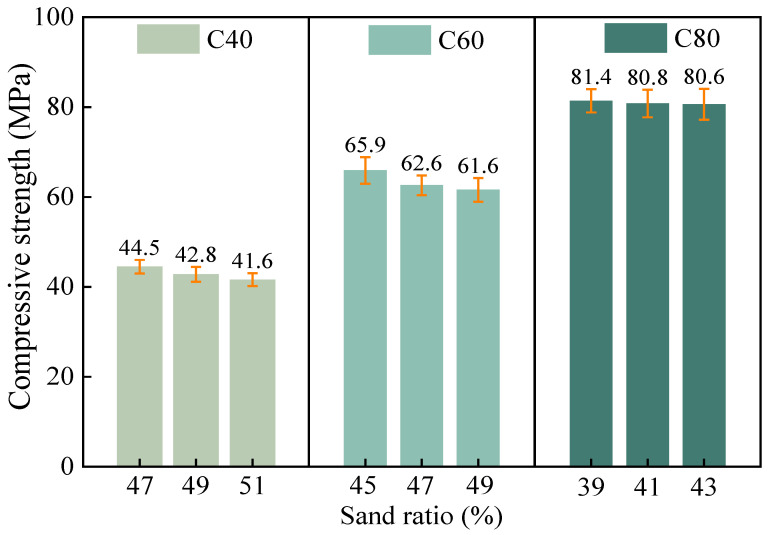
The effect of the sand ratio on the compressive strength of the concrete.

The relationship between the sand ratio and the 28-day compressive strength of the concrete at various strength grades is shown in Figure 9 and indicates a negative correlation between the sand ratio and compressive strength, which is completely opposite to its effect on the workability. As the sand ratio increased, the mass fraction of the coarse aggregates in a unit volume of concrete decreased, which resulted in a reduction in the thickness of the cement paste that envelops the aggregate surfaces. This partially disrupted the closest packing state of the concrete aggregate system. Additionally, the increase in the volume fraction of the mortar reduced the skeleton effect of the coarse aggregates and decreased the frictional resistance between the coarse aggregates.

#### 4.2.3. Effect of Maximum Nominal Aggregate Size on Concrete Compressive Strength

**Figure 10:**
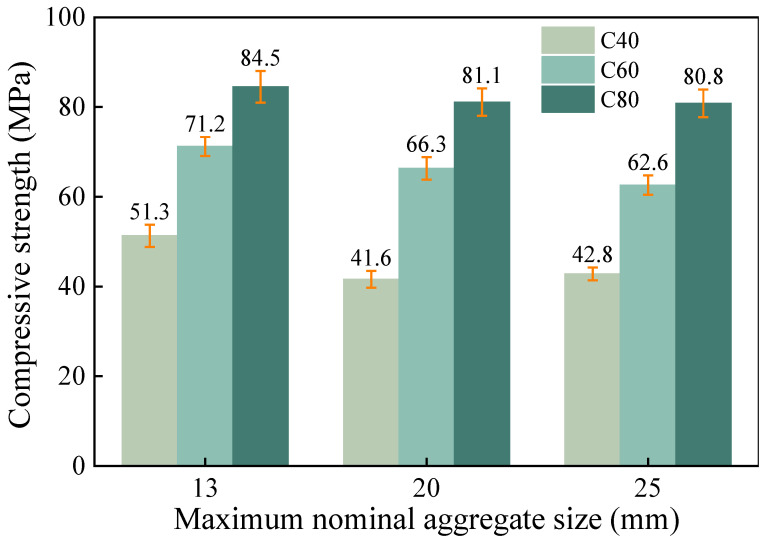
The effect of the maximum nominal aggregate size on the compressive strength of the concrete.

The influence of the maximum aggregate particle size on the compressive strength of the concrete is illustrated in Figure 10. As the maximum nominal particle size of the aggregates decreased, the strength of each concrete group improved to varying degrees. When the maximum nominal particle size was reduced from 25 mm to 20 mm and 13 mm, the strength change rates for the C40 group were −2.8% and 23.3%, respectively; for the C60 group, the strength increased by 5.9% and 13.7%; for the C80 group, it rose by 2.9% and 7.2%. The maximum nominal particle size of the aggregates can influence their specific surface area, which, in turn, affects the water requirement for wetting the unit mass of the aggregate surfaces [58]. As the maximum nominal particle size of the concrete aggregates increased, the water requirement for wetting the aggregate surfaces decreased [60], allowing for a greater amount of water to participate in the hydration of the cement. This resulted in a decrease in the concrete strength as the maximum nominal particle size increased, while maintaining the same water-to-cement ratio. Furthermore, compared to the C80 group with a lower water-to-cement ratio, the mechanical properties of the C60 group concrete were more sensitive to changes in the maximum nominal particle size.

### 4.3. The Relationship Between Concrete Mix Proportion Parameters, Workability, and Strength Grade

**Figure 11:**
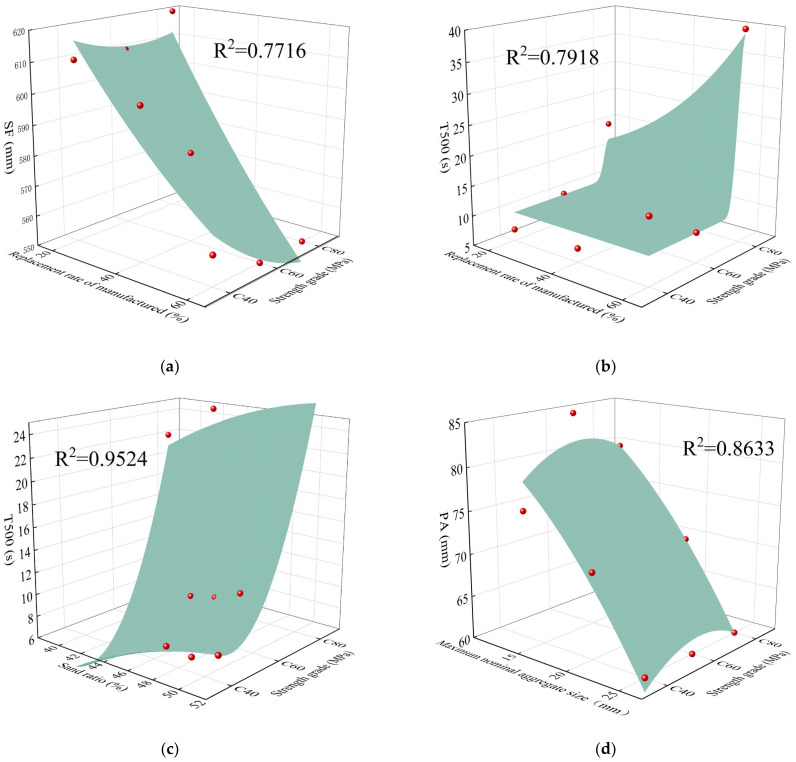
(**a**) SF–fine aggregate replacement rate/strength grade relationship; (**b**) T500–fine aggregate replacement rate/strength grade relationship; (**c**) T500–sand ratio/strength grade relationship; (**d**) PA–maximum nominal aggregate size/strength grade relationship.

To refine the mix design of the MSH-SCC and elucidate the interplay between the mix proportion parameters, self-compacting concrete indicators, and concrete strength grades, this study leveraged statistical methods to model the data. The red dots represented the data points obtained from the concrete’s workability and strength tests conducted in Section 4.1 and Section 4.2. By fitting the data points, a model was constructed in Figure 11. The model correlated the concrete strength with workability indicators such as the slump flow, T500, and PA. The model plots the manufactured sand replacement rate, sand ratio, and maximum nominal aggregate size on the *x*-axis against the strength grades on the *y*-axis.

Figure 11 clearly indicates a substantial decrease in the concrete workability beyond the design strength of the C60 concrete. To optimize the balance between the fill ability, passing ability, and strength in the MSH-SCC, strict control over the replacement rate of the manufactured sand and the maximum particle size of the coarse aggregate is essential in both the design and construction. To ensure the self-compacting performance of the MSH-SCC, it is recommended to opt for a lower replacement rate of manufactured sand and a smaller maximum nominal particle size. Within the range of sand ratios studied, the sand ratio’s influence on the workability was not significant. Given the paucity of research on MSH-SCC preparation, these findings offer a valuable reference for future research endeavors and practical engineering applications.

## 5. Conclusions

In this study, MSH-SCC mixtures with 20% manufactured sand were optimized for the following three strength levels: C40, C60, and C80. The effects of the manufactured sand replacement rate, sand ratio, and maximum nominal aggregate size on the workability, rheology, and strength of the MSH-SCC were analyzed.

- Relative to the initial mix proportions, the optimized mix proportions have markedly enhanced the workability of the concrete, while only experiencing a marginal reduction in the strength, which remains compliant with the specified strength requirements.
- The influence of the mix proportion parameters on the workability of the MSH-SCC varies. To ensure good workability, it was important to reasonably select the replacement rate of the manufactured sand and to recommend a smaller maximum nominal aggregate size. Particularly in high-strength concrete, the MSH-SCC was more sensitive to fluctuations in the maximum nominal size of the coarse aggregates; therefore, special attention should be given to this aspect during the mix design.
- The yield stress of the concrete positively correlates with the manufactured sand replacement and maximum nominal aggregate size, but negatively correlates with the sand ratio. The C80 concrete displayed high static and low dynamic yield stresses. At a 40% manufactured sand replacement rate, the concrete’s plastic viscosity peaks. The MSH-SCC with a maximum aggregate size of 20 mm exhibits a lower viscosity and superior rheological properties.
- Manufactured sand enhances the mechanical strength of the MSH-SCC. However, for the concrete with higher design strengths, the adjustment of the mix proportion parameters has a relatively limited impact on its mechanical properties.
- Relationships were established between the mix proportion parameters, strength grades, and workability indicators to better guide the design of the MSH-SCC mix proportions. This study, however, being limited by the dataset size, suggests future research incorporating larger databases, and exploring MSH-SCC with increased manufactured sand ratios. Additionally, performance tests could be conducted on MSH-SCC with different types and shapes of manufactured sand to provide references for practical applications.

## Figures and Tables

**Table 1:** Chemical composition of the used cementitious materials.

Constituents	Fe_2_O_3_	SiO_2_	Al_2_O_3_	CaO	MgO	SO_3_	TiO_2_	Loss on Ignition
	(%)							
Cement	3.80	22.15	5.63	66.04	0.96	3.05	1.19	2.21
Fly ash	3.24	58.06	31.28	1.90	0.60	0.85	1.30	1.95
Silica fume	0.20	95.26	0.18	1.43	0.20	0.53	—	2.20
Mineral powder	7.00	50.88	19.41	11.19	1.35	0.69	0.69	2.17

**Table 2:** Technical characteristics of the coarse aggregate.

	Apparent Density	Volume Density	Bulk Density	Porosity	Moisture Content
	(g/cm^3^)	(g/cm^3^)	(s)	(%)	(%)
5–10 mm	2.870	2.820	1.70	39.88	0.83
10–25 mm	2.840	2.815	1.71	40.05	0.70

**Table 3:** Technical characteristics of the fine aggregates.

	Apparent Density	Angularity	Crushing Value	Methylene Blue Value	Sand Equivalent	Moisture Content
	(g/cm^3^)	(s)	(%)	(g/kg)	(%)	(%)
MS	2.650	25.16	6.2	1.1	69	2.00
RS	2.610	21.33	11.4	0.2	75	1.42

**Table 4:** Unoptimized mix design of the SCC with 20% manufactured sand.

Strength Grade	Cement	Fly Ash	Silica Fume	Mineral Powder	10–20 mm Crushed Stone	5–10 mm Crushed Stone	River Sand	Manufactured Sand	Water	SP (%)
	kg/m^3^									
C40	160	120	0	120	741	317	613	153	158	1.3
C60	260	87	0	87	723	325	578	145	152	1.6
C80	449	0	50	78	665	364	532	133	129	2.1

**Table 5:** Components of the optimized aggregate synthesis system.

	10–25 mm Crushed Stone (kg/m^3^)	5–10 mm Crushed Stone (kg/m^3^)	River Sand (kg/m^3^)	Aggregate System Density (kg/m^3^)
C40	771	133	731	183
C60	761	224	700	175
C80	664	415	600	150

**Table 6:** Dosage of cementitious materials for the different strength grades of concrete (wt. kg/m^3^).

	Cement	Fly Ash	Mineral Powder	Silica Fume
C40	192	96	96	—
C60	270	90	90	—
C80	426	—	77	50

**Table 7:** V_paste_/V_void_ for concretes of different strength grades.

Strength Grade	V_paste_/V_void_
	%
C40	161
C60	188
C80	195

**Table 8:** Optimized mix design of the SCC with 20% manufactured sand.

Strength Grade	Cement	Fly Ash	Silica Fume	Mineral Powder	10–20 mm Crushed Stone	5–10 mm Crushed Stone	River Sand	Manufactured Sand	Water	SP (%)
	kg/m^3^									
C40	192	96	0	96	771	133	731	183	153	1.3
C60	270	90	0	90	761	224	700	175	142	1.6
C80	426	0	50	77	664	415	600	150	122	2.1

**Table 9:** The workability of the concrete with the original mix proportions and the optimized mix proportions.

		Slump Flow (mm)	T500 (s)	PA (mm)
C40	Unoptimized	575	7.0	75
C60		590	11.3	95
C80		510	43.5	130
C40	Optimized	610	6.9	70
C60		610	10.0	90
C80		620	20.0	80

**Table 10:** The effect of the manufactured sand replacement rate on the workability of the MSH-SCC at various strength grades.

	Water–Binder Ratio	Replacement Rate of Manufactured Sand	Slump Flow	T500	PA
		(%)	(mm)	(s)	(mm)
C40-M20	0.40	20	610	6.9	70
C40-M40		40	600	7.3	80
C40-M60		60	560	16.0	80
C60-M20	0.32	20	610	10.0	90
C60-M40		40	580	8.3	80
C60-M60		60	550	18.0	90
C80-M20	0.22	20	620	20.0	80
C80-M40		40	560	22.0	80
C80-M60		60	550	40.0	80

**Table 11:** The effect of the sand ratio on the workability of the MSH-SCC at various strength grades.

	Water–Binder Ratio	Replacement Rate of Manufactured Sand	Slump Flow	T500	PA
		(%)	(mm)	(s)	(mm)
C40-S47	0.40	47	610	7.5	100
C40-S49		49	610	7.3	70
C40-S51		51	630	8.2	110
C60-S45	0.32	45	600	9.5	90
C60-S47		47	610	10.0	90
C60-S49		49	630	11.0	100
C80-S39	0.22	39	610	22.0	100
C80-S41		41	620	20.0	80
C80-S43		43	640	25.0	90

**Table 12:** The effect of the maximum nominal aggregate size on the workability of the MSH-SCC at various strength grades.

	Water–Binder Ratio	Replacement Rate of Manufactured Sand	Slump Flow	T500	PA
		(%)	(mm)	(s)	(mm)
C40-R13	0.40	13	610	6.9	75
C40-R20		20	590	8.0	70
C40-R25		25	590	6.9	60
C60-R13	0.32	13	610	10.0	85
C60-R20		20	590	4.3	70
C60-R25		25	650	2.7	60
C80-R13	0.22	13	620	20.0	80
C80-R20		20	580	12.5	70
C80-R25		25	570	40.0	60

## Data Availability

The original contributions presented in this study are included in the article. Further inquiries can be directed to the corresponding author.
